# An Unusual Case of Acute Abdomen: Candida-Associated Jejunal Perforation With Pancolitis in an Immunocompetent Patient

**DOI:** 10.7759/cureus.108397

**Published:** 2026-05-06

**Authors:** Pramod D Nichat, Girish D Bakhshi, Pritesh N Joshi, Shraddha A Dhende, Chetan D Rathod, Arpita Arjwani, Umesh V Murkute, Anuj A Shejwalkar, Nikhil M Dalanje, Akanksha N Ahire, Aman Jaiswal

**Affiliations:** 1 General Surgery, Grant Government Medical College and Sir J. J. Group of Hospitals, Mumbai, IND; 2 General Surgery, Grant Government Medical College and Sir J.J. Group of Hospitals, Mumbai, IND

**Keywords:** candida-associated pancolitis, chronic colitis, edge biopsy, gastrointestinal candidiasis, immunocompetant patient, infectious colitis, invasive candidiasis, jejunal perforation, pancolitis, pseudohyphae

## Abstract

Gastrointestinal candidiasis is typically encountered in immunocompromised individuals. Small bowel perforation presenting in association with pancolitis in an immunocompetent patient is exceedingly rare. We report the case of a 67-year-old immunocompetent male patient who presented with acute abdomen and generalized peritonitis. Emergency exploratory laparotomy revealed a jejunal perforation 10 cm distal to the duodenojejunal flexure, which was managed with primary repair and an omental patch. The postoperative course was initially uneventful; however, the patient was readmitted with diarrhea and abdominal pain. Imaging revealed diffuse pancolitis with terminal ileal involvement. Histopathological examination of the jejunal perforation edge biopsy demonstrated fungal elements in the form of pseudohyphae and budding yeast forms consistent with *Candida* species, without evidence of malignancy or granulomatous disease. The patient responded favorably to systemic antifungal therapy with fluconazole. This case highlights a rare presentation of *Candida*-associated jejunal perforation with pancolitis in an immunocompetent patient and underscores the importance of routine histopathological evaluation in hollow viscus perforation and early consideration of fungal etiology in atypical postoperative gastrointestinal presentations.

## Introduction

*Candida *species are common commensals of the gastrointestinal tract but rarely cause invasive disease in immunocompetent hosts. Gastrointestinal candidiasis most commonly affects the esophagus and stomach, while colonic involvement is infrequently reported. When present, fungal colitis is associated with high morbidity and mortality, particularly due to delayed diagnosis. Small bowel perforation secondary to fungal infection is exceedingly rare, with only isolated case reports described in the literature [1].

We present a rare case of *Candida*-associated jejunal perforation with pancolitis in an immunocompetent patient, emphasizing the diagnostic challenges and the role of histopathology and antifungal therapy in management [2].

## Case presentation

A 67-year-old male patient, a resident of Maharashtra, India, presented with a four-day history of generalized abdominal pain that was insidious in onset, progressively worsening, and unrelieved by analgesics. The pain was associated with nausea and loss of appetite. There was no history of vomiting, fever, gastrointestinal bleeding, trauma, weight loss, or prior abdominal surgery. The patient had no known comorbidities such as diabetes mellitus, hypertension, tuberculosis, or other immunosuppressive conditions. Also had no history of consumption of proton pump inhibitors or non-steroidal anti-inflammatory drugs. 

On examination, the patient was conscious and oriented, with mild dehydration. Vital signs revealed tachycardia (102/min) and borderline hypotension (100/70 mmHg). Abdominal examination showed generalized distension, diffuse tenderness, and absent bowel sounds. Digital rectal examination revealed stool staining without blood.

**Figure 1:**
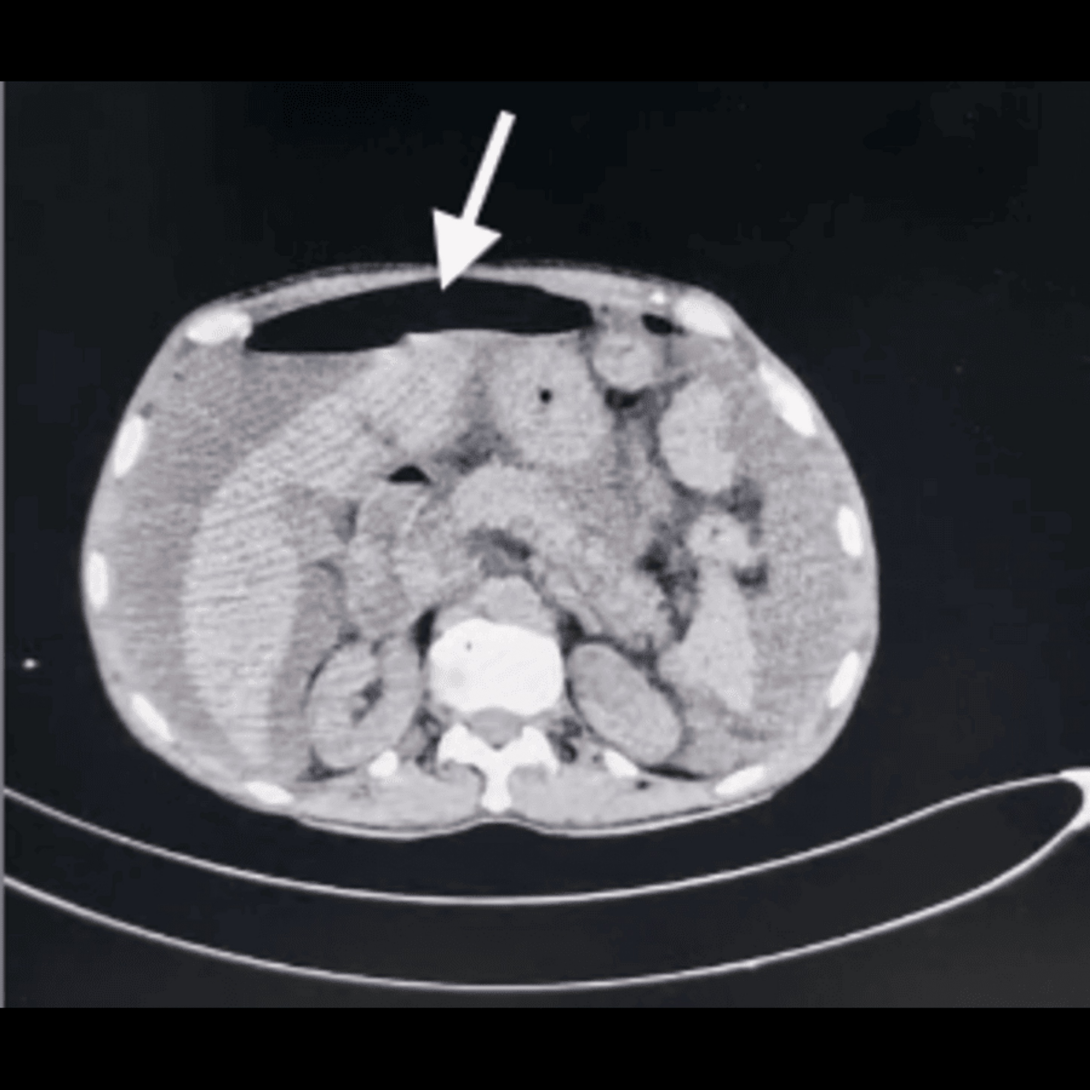
Non-contrast computed tomography of the abdomen demonstrated mild to moderate pneumoperitoneum (white arrow).

Initial laboratory investigations are summarized in Table 1. Serological testing for HIV was negative, and there was no history of steroid use or recent broad-spectrum antibiotic exposure. Arterial blood gas analysis showed no metabolic derangement. Plain radiographs of the chest and abdomen did not reveal free air. Non-contrast computed tomography of the abdomen demonstrated mild to moderate pneumoperitoneum (Figure 1).

**Table 1:** Laboratory Investigations at Initial Presentation

Parameter	Patient Value	Reference Range
Haemoglobin	10.8	13–17 g/dL
Total leukocyte count	8500 /mm³	4,000–11,000 /mm³
Neutrophils	70%	40–75%
Platelet count	2.85 ×10⁵/mm³	1.5–4.0 ×10⁵/mm³
Serum creatinine	1.1 mg/dL	0.6–1.2 mg/dL
Serum sodium	137 mEq/L	135–145 mEq/L
Serum potassium	3.8 mEq/L	3.5-5.0 mEq/L
Human immunodeficiency virus (HIV)/ hepatitis B surface antigen (HBsAg)/ hepatitis C virus (HCV)	Non reactive	

**Figure 2:**
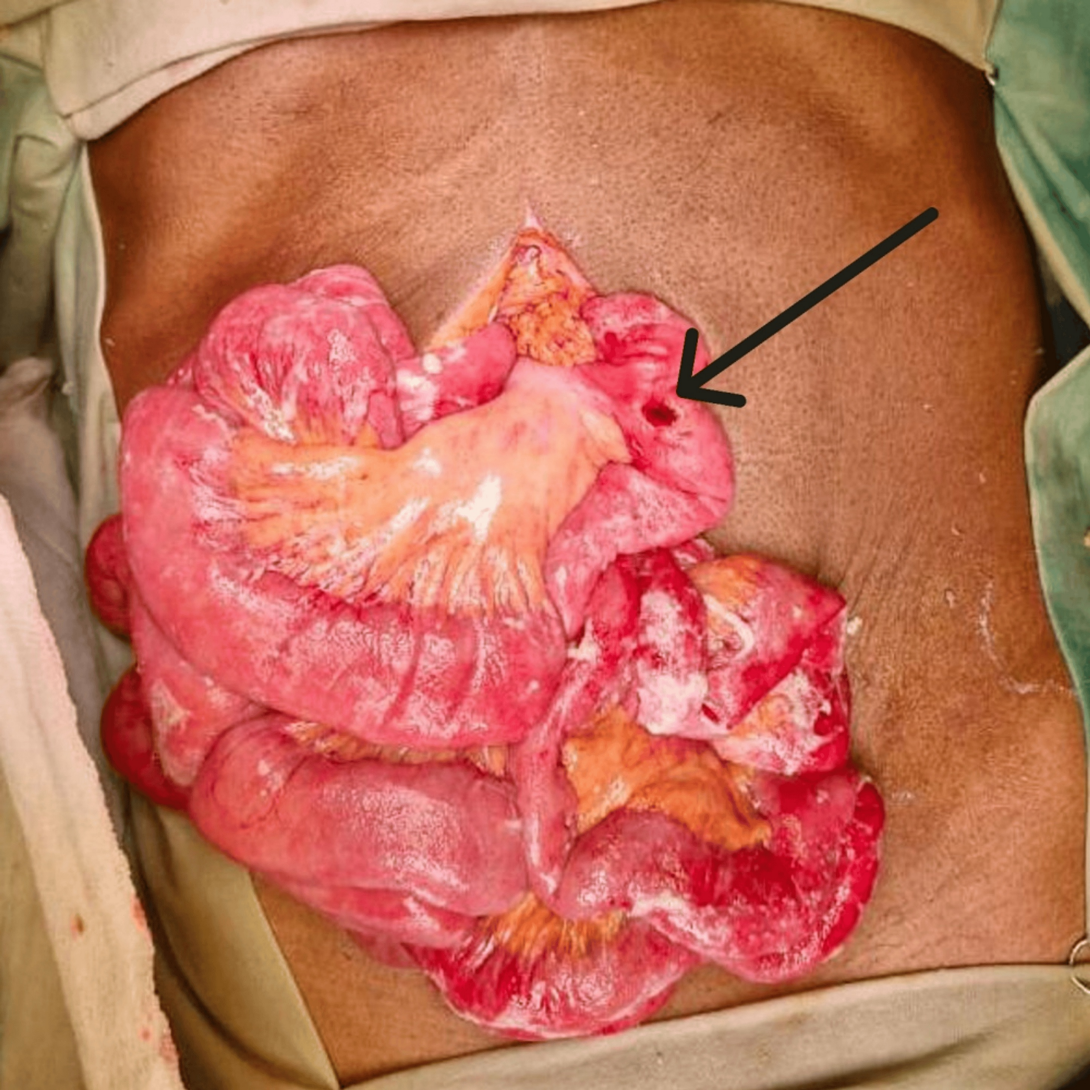
Intraoperative findings during the emergency exploratory laparotomy; jejunal perforation (black arrow) located approximately 10 cm distal to the duodenojejunal flexure.

After adequate resuscitation, the patient underwent emergency exploratory laparotomy. Approximately 1.5 liters of bile-stained peritoneal fluid was evacuated. A 1 × 0.5 cm perforation with ragged edges was identified on the jejunum, approximately 10 cm distal to the duodenojejunal flexure (Figure 2). The remainder of the bowel was unremarkable, with no evidence of adhesions or lymphadenopathy. Primary repair of the perforation was performed using polydioxanone sutures, reinforced with an omental patch, followed by thorough peritoneal lavage and drain placement.

Patient was continued on the same injectable antibiotics that were started on admission (IV ceftriaxone 1gram 12 hourly and IV metronidazole 100 mL eight hourly). Postoperative recovery was initially uneventful. The patient was discharged on postoperative day 7 with advice for follow-up.

Readmission and further evaluation

Three weeks from the date of discharge, the patient presented with four days of loose stools (4-5 episodes/day) and abdominal pain. On examination, he was vitally stable with a healed midline scar and mild generalized abdominal tenderness. Laboratory investigations remained within normal limits. Stool examination showed inflammatory changes with positive occult blood; testing for *Clostridium difficile* toxin was negative.

**Figure 3:**
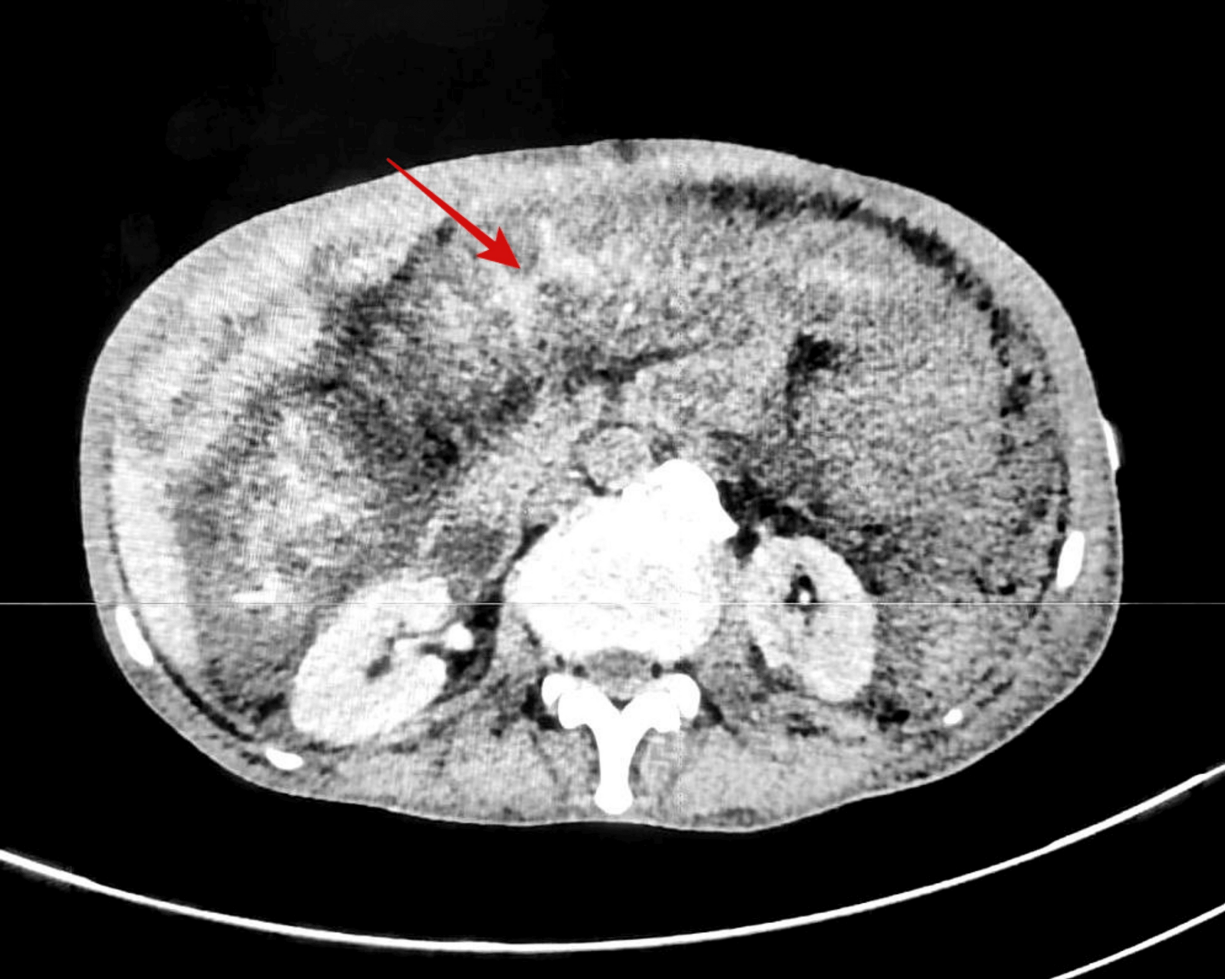
Contrast-enhanced computed tomography of the abdomen demonstrating diffuse circumferential mural thickening of the colon with mucosal hyperenhancement and submucosal edema producing the characteristic "accordion sign" (red arrow), suggestive of pancolitis.

Ultrasonography and contrast-enhanced computed tomography of the abdomen revealed diffuse circumferential mural thickening of the entire colon and terminal ileum, with mucosal hyperenhancement and submucosal edema producing an “accordion sign,” consistent with pancolitis (Figure 3) [3].

**Figure 4:**
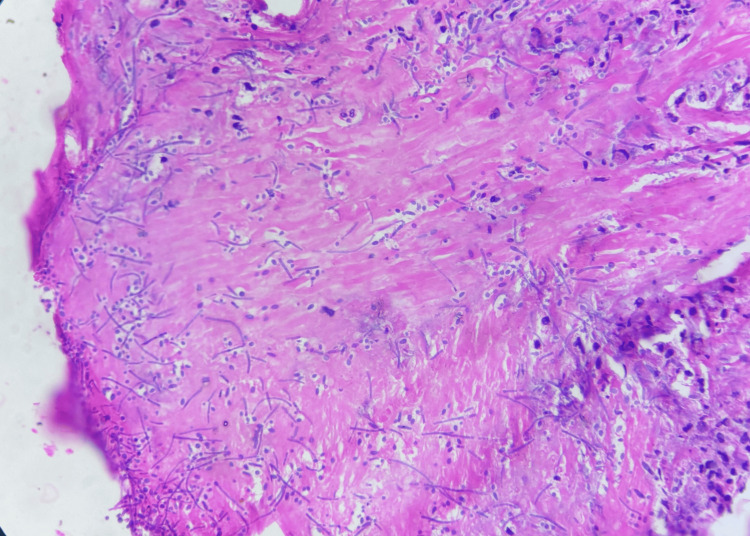
Histopathological examination of the jejunal perforation edge biopsy: hematoxylin and eosin–stained section showing fibro-collagenous tissue with mixed inflammatory infiltrate and fungal elements in the form of pseudohyphae and budding yeast forms (low power view).

**Figure 5:**
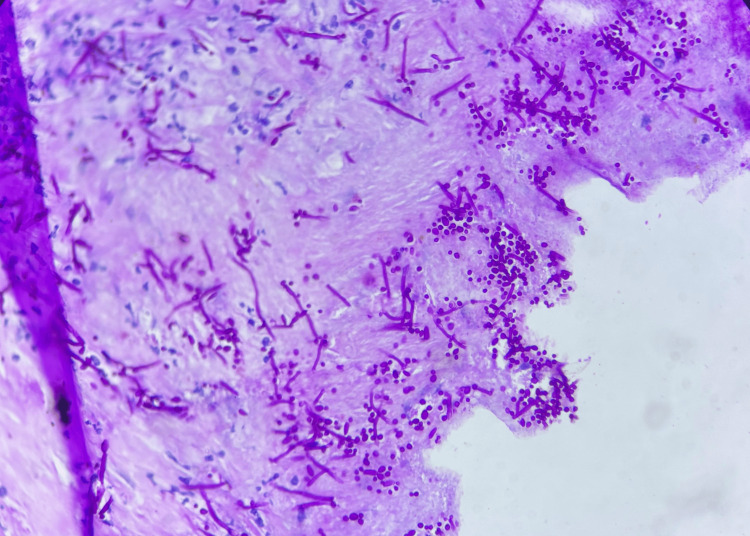
Histopathological examination of the jejunal perforation edge biopsy: periodic acid–Schiff (PAS) stain highlighting fungal pseudohyphae and yeast forms consistent with Candida species (high-power view).

Histopathological examination of the jejunal perforation edge biopsy obtained during index surgery demonstrated fibrocollagenous tissue with mixed chronic inflammatory infiltrate and fungal elements in the form of pseudohyphae and budding yeast forms, suggestive of *Candida *species (Figures 4, 5). No evidence of malignancy, granuloma, or atypia was observed.

The patient was managed conservatively with bowel rest, intravenous fluids, and systemic antifungal therapy (oral fluconazole for 14 days). Empirical oral vancomycin was initiated but discontinued after 48 hours when *Clostridium difficile* infection was excluded. The patient showed marked symptomatic improvement within five days and was discharged in stable condition.

Patient timeline

The patient presented on day 0 (index presentation). Surgery was performed on day 1. The patient was discharged on day 8. The patient was readmitted on day 29, followed by discharge on day 34.

## Discussion

Invasive gastrointestinal candidiasis is typically associated with immunosuppression, prolonged antibiotic use, corticosteroid therapy, or major abdominal surgery. Small bowel perforation is rare, and delayed presentation with pancolitis is exceptional, particularly in immunocompetent individuals [4].

The pathogenesis of *Candida*-associated gastrointestinal disease involves disruption of mucosal barriers and alteration of normal gut flora, allowing fungal overgrowth and invasion [5].

The diagnosis of fungal colitis is challenging due to nonspecific clinical and radiological findings that often mimic inflammatory bowel disease or pseudomembranous colitis. Histopathological demonstration of fungal elements remains crucial for diagnosis. Early recognition and prompt antifungal therapy are essential, as delayed treatment is associated with high mortality rates reported in the literature [6].

While bowel perforation associated with candidiasis has been reported previously, such cases are exceedingly rare and often occur in immunocompromised hosts. As described earlier, bowel perforation associated with candidial pancolitis in immunocompetent individuals remains extremely limited. This underscores the unusual nature of the present case and highlights the diagnostic challenge posed by such atypical presentations.

## Conclusions

Jejunal perforation occurring in the setting of *Candida*-associated pancolitis in an immunocompetent individual is extremely rare, with very limited documentation in the existing literature. Reporting such atypical presentations contributes to the growing clinical database and may aid clinicians in considering fungal etiologies in unexplained gastrointestinal perforations.

This case underscores the importance of routine histopathological examination of perforation edges, vigilant postoperative follow-up, and consideration of fungal etiology in patients with atypical gastrointestinal presentations. Early initiation of appropriate antifungal therapy can lead to favorable outcomes. We report this case to raise awareness of *Candida *as a potential etiology of small bowel perforation in immunocompetent hosts and to emphasize diagnostic strategies.
